# Exploring the Relationship Between Students’ Language Learning Curiosity and Academic Achievement: The Mediating Role of Foreign Language Anxiety

**DOI:** 10.3390/bs15081133

**Published:** 2025-08-20

**Authors:** Honggang Liu, Tong Li, Hongying Zheng, Yang Li, Jiqun Fan

**Affiliations:** 1 School of Foreign Languages, Soochow University, Suzhou 215006, China; liuhonggang@suda.edu.cn; 2 Suzhou Pingjiang Middle School, Suzhou 215021, China; 3 School of Foreign Languages, Sichuan Normal University, Chengdu 610021, China; 4 School of Foreign Languages, Jilin University of Architecture and Technology, Changchun 130114, China; 5 School of Foreign Languages, Huainan Normal University, Huainan 232063, China

**Keywords:** language learning, curiosity, anxiety, academic achievement, junior high school student

## Abstract

Curiosity and anxiety are critical emotional factors influencing language learning; yet, existing studies often overlook their combined effects and the mechanisms through which they shape academic achievements. This study aimed to explore the relationships among language learning curiosity, foreign language anxiety, and academic achievement among junior high school students. A total of 870 students from southeastern China, including 7 selected for semi-structured interviews, participated in the study. Quantitative data were analyzed through SPSS and AMOS with structural equation modeling, while qualitative data were examined using thematic analysis in NVivo to identify profile-based patterns. The findings revealed that students showed relatively high curiosity and moderate anxiety in English language learning. Consistent with our hypotheses, curiosity positively predicted academic achievement, while anxiety had a negative effect. Foreign language anxiety partially mediated the relationship between curiosity and academic performance. Qualitative data supported these findings, showing that curiosity fostered engagement and reduced anxiety in real learning contexts. These findings support the control-value theory of academic emotions and offer theoretical and practical implications for emotional regulation in language education.

## 1. Introduction

As the global demand for English proficiency intensifies, language education in exam-oriented systems like China has increasingly emphasized academic achievement (Wang et al., 2024; Yu & Luo, 2025). Junior high school students, positioned at a critical stage of cognitive and emotional development, often experience strong affective reactions during the learning process (Yao et al., 2021). Among these, curiosity and anxiety emerge as particularly influential emotions that shape engagement and performance in second language learning (Fraschini & Tao, 2023; Hong et al., 2022; Liu et al., 2025a). Curiosity encourages exploration and persistence in the face of challenges, whereas anxiety can lead to avoidance and impaired processing (Dewaele & Botes, 2025; Liu et al., 2025b; Mahmoodzadeh & Khajavy, 2019). Recognizing the emotional complexity of adolescent learners, researchers have begun to explore the role of emotions in English language learning (Liu et al., 2025c; Pavelescu, 2019). However, the ways in which curiosity and anxiety interact and influence academic success during this transitional stage of education remain underexplored. A deeper understanding of these emotional dynamics may provide valuable insights for improving student motivation, emotional well-being, and academic outcomes in language classrooms (Zhang et al., 2024).

Prior studies have investigated language learning curiosity and foreign language anxiety independently, consistently linking curiosity with enhanced learning motivation and anxiety with reduced performance (Fraschini & Tao, 2023; Hong et al., 2022). Nonetheless, much of this research has been conducted with university students or adults (Fraschini & Tao, 2023; Mohammadi et al., 2013), offering limited insight into younger learners in high-pressure East Asian environments. Furthermore, few studies have examined the interplay between these emotions or their combined effects on academic achievement in language learning. There is also a lack of integrative frameworks that explain the psychological mechanisms connecting emotion and learning.

To address these gaps, the present study adopts the control-value theory of academic emotions (Pekrun, 2006) to examine the relationships among language learning curiosity, foreign language anxiety, and English academic achievement in Chinese junior high school students. To explore these relationships, the study adopts a mixed-methods design, combining quantitative data from validated questionnaires with qualitative insights from semi-structured interviews. This research presents two key innovations. First, it extends the application of the control-value theory of achievement emotions to early adolescent language learners, a group underrepresented in the current literature. Second, it investigates the dual roles of curiosity and anxiety within a single model to reflect emotional complexity in classroom settings. This study aims to offer meaningful contributions by enhancing the theoretical understanding of emotional–cognitive interactions in second language acquisition and providing evidence-based guidance for developing emotionally responsive pedagogy in exam-oriented educational contexts.

## 2. Literature Review

### 2.1. Curiosity, Anxiety, and Academic Achievements in English Language Learning

Curiosity is conceptualized as an epistemic drive to seek novel information that motivates exploratory behavior (Berlyne, 1978; Litman & Jimerson, 2004). Building on this, Litman (2007) proposed that curiosity can emerge from an intrinsic desire to resolve uncertainty, often accompanied by tension and a strong need to acquire information. Loewenstein (1994) argued that curiosity arises either from the anticipated pleasure of learning or from the discomfort caused by information gaps, reflecting two distinct motivational pathways. Mahmoodzadeh and Khajavy (2019) defined language learning curiosity as an inquiry-driven desire to learn and use a foreign language. They identified two key dimensions: curiosity as a feeling of interest and curiosity as a feeling of deprivation, reflecting learners’ enjoyment of exploration and their urge to resolve knowledge gaps. Building on this framework, researchers have examined learner curiosity from multiple angles, including affective engagement (Vracheva et al., 2020), cognitive inquiry (Wu & Wu, 2020), behavioral indicators (Spielberger & Starr, 2012), instructional context (Peterson, 2020), and cultural variations (Bluemke et al., 2023). These findings collectively emphasize that language learning curiosity is a multifaceted psychological construct that plays a central role in sustaining learners’ engagement and promoting long-term academic growth in second language education.

Anxiety is defined as a unique, negative emotional experience related to language learning that stems from learners’ self-perceptions and the challenges of acquiring a new language (Horwitz et al., 1986). It includes both cognitive components like worry (Eysenck, 1979; Borkovec, 1985) and affective symptoms such as tension and nervousness (Spielberger, 1971). Scholars have further conceptualized it from psychological (Derakhshan & Fathi, 2024; Dewaele et al., 2024, 2025a, 2025b; Liu et al., 2023), situation-specific (Liu et al., 2024), and social perspectives (Oxford, 1993). MacIntyre and Gardner (1994) categorized it into input, processing, and output anxiety, highlighting how it affects each stage of language comprehension and production. Researchers have examined foreign language anxiety through five main themes: psychological reactions (Dryden et al., 2021), situation-specific contexts (Hsu et al., 2023), and social influences like fear of negative evaluation (Yılmaz & De Jong, 2024). Additional focus has been placed on cognitive processing stages and the functional impact of anxiety, distinguishing between its facilitating and debilitating effects on learning (Chen, 2022). These perspectives collectively illustrate the multidimensional nature of foreign language anxiety and its significant implications for language education.

Academic achievement refers to the measurable performance outcomes that reflect a student’s learning progress and mastery of educational content (Steinmayr et al., 2014). It is typically assessed through grades, standardized test scores, and classroom evaluations, and it is considered a key indicator of educational success (York et al., 2015). According to Pekrun (2006), academic achievement is not only shaped by cognitive ability but also by affective factors, such as emotions, motivation, and self-regulation. In second language acquisition contexts, academic achievement in English language learning often includes proficiency in reading, writing, listening, and speaking, as well as students’ ability to apply language skills in academic tasks (Ardasheva et al., 2012; Li et al., 2022). Researchers have increasingly emphasized the role of emotional and motivational variables in influencing academic outcomes, highlighting that high achievement often results from the interaction of cognitive engagement, emotional well-being, and supportive learning environments (Camacho-Morles et al., 2021; Feraco et al., 2023). Thus, academic achievement represents a complex construct shaped by both intellectual and emotional experiences.

### 2.2. Theoretical Foundation: Control-Value Theory of Achievement Emotions

The control-value theory of academic emotions, proposed by Pekrun (2006), provides a structured framework for analyzing learners’ emotional responses in academic settings. According to this theory, achievement emotions stem from two primary appraisals: perceived control over academic tasks and the subjective value attached to them (Pekrun & Perry, 2014). Control appraisals concern a student’s confidence in handling learning demands, while value appraisals refer to the personal significance or usefulness of those tasks. These appraisals work together to influence emotional outcomes, such as enjoyment, anxiety, or boredom. Additionally, the theory accounts for contextual and personal factors, such as instructional practices, social expectations, autonomy support, and personality traits like self-efficacy and goal orientations (Pekrun, 2006; Yang et al., 2021; Yue et al., 2024). As a result, the theory captures the complex interplay between cognition, emotion, and learning environment in shaping students’ academic behaviors and emotional experiences.

The control-value theory of academic emotions offers a valuable framework for interpreting the emotional dynamics of language learning among junior high school students. Its emphasis on control and value appraisals aligns closely with the core constructs of this study, particularly language learning curiosity and foreign language anxiety. Curiosity can shape how students evaluate their control over learning tasks and the value they assign to language learning, both of which are central to the development of achievement emotions (Pekrun & Perry, 2014). In this context, students with high curiosity are more likely to perceive English language learning as meaningful and manageable, which reduces anxiety and enhances performance. By incorporating both cognitive and emotional variables into a unified model, the theory provides a coherent explanation for how motivational traits influence emotional states and academic outcomes. Therefore, it serves as a suitable theoretical lens to explore how foreign language anxiety mediates the relationship between students’ curiosity and their academic achievement in English learning.

### 2.3. The Hypothesized Model

**Figure 1:**
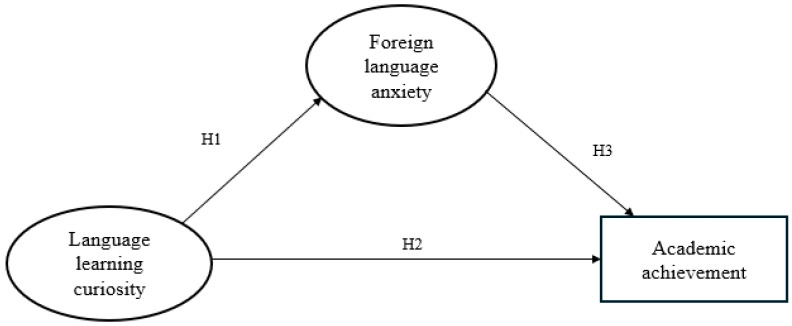
The hypothesized model.

In the field of second language acquisition, increasing attention has been devoted to the psychological dimensions that underlie learner behavior and performance. This study is informed by the control-value theory of academic emotions and of achievement emotions, which provides a comprehensive framework for examining the interplay between emotional experiences and learning processes. Within this framework, cognitive–affective constructs such as curiosity and anxiety are conceptualized as interacting factors that influence learners’ engagement, persistence, and academic outcomes (see Figure 1). Building on these theoretical foundations, the present study proposes the three hypotheses presented below.

Curiosity in language learning may help reduce learners’ feelings of tension and apprehension. This is because curiosity encourages engagement and confidence, which are often disrupted by anxiety. Prior research has identified a negative relationship between curiosity and anxiety in educational settings (Hong et al., 2022; Kashdan & Roberts, 2004; Taheri et al., 2024). Based on this, we propose the following hypothesis.

**H1.**  *Students’ curiosity can negatively and directly predict anxiety.*

Curious learners are more likely to seek knowledge actively and persist through academic challenges. This tendency supports deeper learning and leads to better academic performance. Studies have shown that curiosity contributes to higher academic achievement by promoting effort and effective learning strategies (Eren & Coskun, 2016; Hong et al., 2022; Tang & Salmela-Aro, 2021). Based on this, we propose the following hypothesis.

**H2.**  *Students’ curiosity can positively and directly predict academic achievement.*

Anxiety in language learning often interferes with performance by affecting attention, memory, and motivation. As a result, students with higher anxiety tend to perform worse in academic tasks. Numerous studies have confirmed a strong negative correlation between language anxiety and academic achievement (Botes et al., 2020; Dewaele & Ergün, 2020; Xu et al., 2022). Based on these findings, we propose the following hypothesis.

**H3.**  *Students’ anxiety can negatively and directly predict academic achievement.*

## 3. Method

This study aims to examine the relationships among junior high school students’ language learning curiosity, anxiety, and academic achievement in English. Adopting an explanatory sequential mixed-methods design, the research first employed quantitative survey data to explore general patterns, followed by qualitative interviews to deepen the interpretation of the findings. This approach allows for a comprehensive understanding of both statistical relationships and learners’ subjective experiences.

### 3.1. Research Questions

The study addresses the following three research questions:

RQ1: What are the profiles of junior high school students’ curiosity and anxiety in English language learning?

RQ2: What are the relationships among junior high school students’ curiosity, anxiety, and academic achievement in English language learning?

RQ3: What is the mediating effect of English language learning anxiety on the relationship between junior high school students’ curiosity and academic achievement in learning English?

### 3.2. Research Context and Participants

In China, junior high school typically includes students aged 12–15 and covers Grades 7–9. During this stage, English is a core academic subject and one of the main components assessed in the high-stakes senior high school entrance examination (Zhongkao). The exam-oriented nature of the education system places heavy emphasis on grammar, vocabulary, and test-taking skills, often at the expense of communicative competence and learner autonomy. As a result, students frequently experience elevated pressure, which can trigger emotional responses such as anxiety and reduce intrinsic interest in language learning. At the same time, this stage coincides with key cognitive and emotional developmental changes, making students particularly sensitive to motivational and affective factors. Investigating curiosity and anxiety in this context is essential to understanding how students’ emotional experiences influence their academic achievement in English and how supportive pedagogical approaches might foster both emotional well-being and academic success.

This study surveyed 870 junior high school students from southeastern China using convenience sampling at the class level. The sample consisted of 422 males and 448 females, including 355 first-year students, 296 second-year students, and 219 third-year students. All participants were enrolled in public schools and had received formal instruction in English as a foreign language. For the qualitative phase, seven students were purposefully selected through maximum variation sampling based on grade level and gender to ensure diverse perspectives. Their demographic and psychological profiles, including scores on language learning curiosity and foreign language anxiety, are presented in Table 1.

### 3.3. Instruments

#### 3.3.1. Language Learning Curiosity Scale

Students’ curiosity in English language learning was measured using the Language Learning Curiosity Scale (see Appendix A.1), developed by Mahmoodzadeh and Khajavy (2019). This instrument contains 11 items rated on a 6-point Likert scale (1 = strongly disagree, 6 = strongly agree), designed to capture learners’ intrinsic motivation and desire to explore linguistic knowledge. The scale includes two conceptual components: curiosity driven by interest and curiosity driven by deprivation. During the pilot study, two items were removed due to poor factor loadings, and minor model adjustments were made. Confirmatory factor analysis supported a unidimensional structure with good model fit (χ^2^/df = 3.552, GFI = 0.955, CFI = 0.973, RMSEA = 0.077). The internal consistency of the revised scale was high (Cronbach’s α = 0.926), indicating that it is a reliable instrument for assessing English language learning curiosity among junior high school students.

#### 3.3.2. Foreign Language Classroom Anxiety Scale

Foreign language anxiety was assessed using a short-form version of the Foreign Language Classroom Anxiety Scale(see Appendix A.2), adapted from Botes et al. (2020). The original instrument included 8 items measuring learners’ anxiety during an English class, particularly related to speaking, peer comparison, and uncertainty in communication. All items were rated on a 6-point Likert scale. Following the pilot study, two reverse-scored items were excluded due to low reliability, and one additional item was removed after confirmatory factor analysis. The final version contained five items forming a single-factor structure. This revised model demonstrated excellent fit indices (χ^2^/df = 0.297, GFI = 0.999, CFI = 1.000, RMSEA = 0.000), with satisfactory internal consistency (Cronbach’s α = 0.859). The scale effectively captured students’ affective reactions in English language learning contexts and provided a concise yet psychometrically sound measure of language-related anxiety for adolescent learners.

#### 3.3.3. Academic Achievement

Students’ academic achievement in English was measured using scores from the Regional Proficiency Assessment, a standardized end-of-year examination administered by the Provincial Department of Education. The test is designed to ensure consistency across schools in terms of content coverage, item difficulty, and scoring criteria. To eliminate potential bias caused by local grading policies, raw test scores (out of 120) were converted into standardized z-scores using IBM SPSS Statistics. These standardized values, used for analysis, reflect students’ relative performance within the entire sample. This approach enhanced comparability and statistical validity when evaluating the impact of psychological variables on English learning outcomes. The standardized scores were labeled “Academic Achievement” and served as the primary indicator of language learning achievement in the structural equation modeling analysis.

### 3.4. Data Collection

Data were collected in two sequential phases: an initial questionnaire phase followed by semi-structured interviews. In the first phase, an online questionnaire was distributed via Wenjuanxing, a widely used Chinese survey platform, between June and September 2024. Participants provided informed digital consent prior to participation and were presented with a brief overview of the study on the first page of the survey. They were assured that the data would be used solely for research purposes, that their responses would have no impact on their academic standing, and that no personally identifiable information would be collected. In the second phase, seven students participated in one-on-one semi-structured interviews conducted in Chinese. All interviews were conducted in Chinese and transcribed verbatim by the researcher. The thematic analysis was carried out in Chinese using the full transcripts. For reporting purposes, representative excerpts were then translated into English for inclusion in the manuscript. Participants were recruited through a voluntary question at the end of the survey inviting students to provide contact information for follow-up. Of the fifteen students initially contacted across different schools and grade levels, four withdrew, and thematic saturation was reached by the ninth interview. Thus, seven interviews were selected for the final analysis based on variation in curiosity and anxiety profiles, as well as demographic diversity. With participants’ consent, all interviews were audio-recorded, transcribed verbatim, and translated into English for thematic analysis. Pseudonyms were used for all interviewees to protect their identities.

### 3.5. Data Analysis

Data were analyzed using SPSS 26.0 and AMOS 24.0. Mahalanobis distance was applied to identify and exclude multivariate outliers. To address potential common method bias associated with self-report measures, Harman’s single-factor test was conducted. Descriptive and correlation analyses were then performed to provide an overview of the data and examine initial relationships among variables. Structural equation modeling was used to test the hypothesized model, which examined both the direct and indirect effects of language learning curiosity on academic achievement through foreign language anxiety. To control for Type I error inflation resulting from multiple comparisons, the Bonferroni correction was applied, setting the adjusted significance level at *p* < 0.001. The significance of the mediating effect was assessed using bootstrapping with 5000 resamples to generate a bias-corrected 95% confidence interval; mediation was considered statistically significant if the interval did not contain zero. Model fit was evaluated using multiple indices, including the chi-square to degrees of freedom ratio (x^2^/df), goodness-of-fit index (GFI), adjusted goodness-of-fit index (AGFI), comparative fit index (CFI), root mean square error of approximation (RMSEA), and root mean square residual (RMR). The following thresholds were used as benchmarks: x^2^/df ≤ 8; GFI ≥ 0.90; AGFI ≥ 0.90; CFI ≥ 0.90; RMSEA ≤ 0.08; RMR ≤ 0.10 (Kline, 2016).

## 4. Results

### 4.1. Profiles of Junior High School Students’ Curiosity and Anxiety in English Language Learning

Language learning curiosity among junior high school students showed a relatively high level, with a mean score of 3.96 (*SD* = 1.13) on a 6-point Likert scale. This score exceeds the scale midpoint, indicating that students generally displayed strong motivation to explore and engage with English beyond surface-level learning. This quantitative trend was supported by qualitative data. For example, Student Zhong expressed a strong sense of curiosity, stating that she frequently sought out English-language movies, vocabulary from subtitles, and real-life applications through participation in English corners. These behaviors reflect not only interest but also a desire to deepen learning beyond classroom requirements.

“I’m always on the lookout for additional English language learning resources and activities… engaging with English in a fun and interactive way makes the learning process more enjoyable and less like a chore.”

(Student Zhong)

Foreign language anxiety was reported at a moderate level across the sample, with a mean of 3.57 (*SD* = 1.21). This score, which lies slightly above the midpoint of the 6-point scale, indicates that students experienced some tension or discomfort in the context of English language learning, though not to a severe degree. This pattern was also evident in students’ interview reflections. Student Chen described notable anxiety during English exams and collaborative tasks, particularly due to fear of forgetting grammar rules or disappointing team mates.

“I get a bit nervous when we have English exams… it’s like my mind goes blank sometimes when I’m under pressure… I worry about letting my teammates down if my part isn’t perfect.”

(Student Chen)

### 4.2. Relationships Among Junior High School Students’ Curiosity, Anxiety, and Academic Achievement in English Language Learning

The second research question explored the relationships among language learning curiosity, foreign language anxiety, and academic achievement. To address this question, Pearson correlation analysis was conducted, which revealed statistically significant correlations among all three variables. The strongest relationship was observed between language learning curiosity and academic achievement (r = 0.423, *p* < 0.01), representing a moderate positive correlation. A moderate negative correlation was found between foreign language anxiety and academic achievement (r = −0.272, *p* < 0.01), suggesting that higher anxiety is associated with lower performance. The weakest relationship was found between foreign language anxiety and language learning curiosity (r = −0.145, *p* < 0.01), indicating a minimal negative correlation.

### 4.3. The Mediating Effect of English Language Learning Anxiety Between Junior High School Students’ Curiosity and Academic Achievement in English Learning

The structural equation model investigated the relationships among language learning curiosity, foreign language anxiety, and academic achievement. After modifications, the model demonstrated a satisfactory fit with the data: GFI = 0.935, AGFI = 0.910, CFI = 0.960, RMSEA = 0.063, and RMR = 0.119. These values meet commonly accepted criteria, indicating that the model adequately represented the observed data.

**Figure 2:**
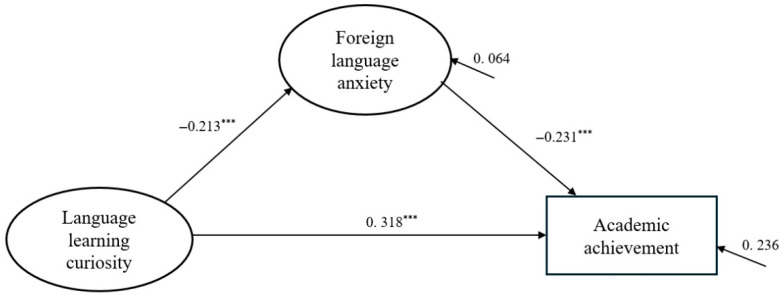
The final model. Note: *** *p* < 0.001.

As shown in Table 2 and Figure 2, all structural paths were statistically significant. Language learning curiosity had a significant negative effect on foreign language anxiety (β = −0.213, *p* < 0.001) and a significant positive effect on academic achievement (β = 0.381, *p* < 0.001). Foreign language anxiety negatively predicted academic achievement (β = −0.231, *p* < 0.001). The model explained 4.6% of the variance in anxiety and 23.6% of the variance in academic achievement. These results support a partial mediation model in which foreign language anxiety mediates the relationship between curiosity and academic performance.

To further examine the mediating role of foreign language anxiety, bootstrapping analysis was conducted with 5000 resamples to assess the indirect, direct, and total effects of language learning curiosity on academic achievement (see Table 3). The results indicated that the indirect effect of curiosity on academic achievement through anxiety was statistically significant (β = 0.049), with the bias-corrected 95% confidence interval [0.019, 0.110] and the percentile 95% confidence interval [0.017, 0.105], both excluding zero. The direct effect of curiosity on academic achievement remained significant (β = 0.381), with confidence intervals well above zero. The total effect, combining both direct and indirect pathways, was β = 0.430. These findings confirm that foreign language anxiety partially mediates the relationship between language learning curiosity and academic achievement, suggesting that while curiosity directly enhances performance, it also indirectly contributes by reducing anxiety levels.

#### 4.3.1. Direct Effect of Curiosity on Academic Achievement

Language learning curiosity was found to be a significant direct predictor of academic achievement in English language learning. Specifically, students who demonstrated higher levels of curiosity were more likely to perform better academically, as their intrinsic motivation led to deeper engagement with learning materials. This effect was not merely motivational but translated into observable academic success. One compelling example comes from Student Yan, whose personal experience vividly illustrates this connection. Initially struggling with Shakespeare’s archaic language, he became intrigued by the historical features of Early Modern English. Driven by genuine curiosity rather than external pressure, he sought out supplementary materials, such as videos and articles, to deepen his understanding. As a result, his interpretation of a monolog was praised by his teacher, reflecting both improved comprehension and performance. His experience underscores how curiosity-driven exploration can enhance learning outcomes by transforming unfamiliar content into a source of academic strength.

“When we started studying Shakespeare in class, it was all old English and super confusing at first. But I was so curious about how people talked back then, so I started reading up on it at home. I even watched some videos online to hear how the language sounded. It wasn’t just about getting the grade; I really wanted to understand it. And guess what? My teacher noticed. She said I had one of the best interpretations of a monologue we read. It felt awesome to connect with the text and do well on the assignment because I actually sought out to learn more.”

(Student Yan)

#### 4.3.2. Direct Effect of Anxiety on Academic Achievement

Foreign language anxiety was found to have a direct and significant negative effect on students’ academic achievement. This finding suggests that anxiety functions not only as an emotional response but also as a cognitive barrier that undermines students’ ability to perform well in academic settings. When students experience persistent anxiety, their mental resources may be diverted toward self-monitoring, fear of failure, or overthinking, which reduces their capacity to focus, process information efficiently, and apply previously learned knowledge. A clear example comes from Student Feng. Although she had practiced her speaking skills extensively for an English oral presentation, she experienced intense anxiety when standing in front of the class. Her mind went blank, her pronunciation became unclear, and she forgot key phrases she had rehearsed. As a result, her presentation was disorganized, and her score dropped significantly despite her prior preparation. This case illustrates how foreign language anxiety can override preparation and interfere with language production and performance. It also highlights the importance of emotional regulation strategies for students facing communication-related stress in second language learning.

“I had practiced my English speech over and over, but when I stood in front of everyone, I froze. I couldn’t remember what to say, and I kept mispronouncing words. It felt like my brain shut off. I only finished half of what I had planned. Afterward, I was so disappointed because I knew the content. Now I try to practice with a small group first to feel less nervous.”

(Student Feng)

#### 4.3.3. Direct Effect of Curiosity on Foreign Language Anxiety

Language learning curiosity was found to have a direct and negative predictive effect on foreign language anxiety. This suggests that students who exhibit stronger curiosity in language learning are less likely to experience anxiety in classroom contexts. Rather than viewing English solely as a test subject, these students engage with it as a meaningful and enjoyable domain, which reduces apprehension and fosters greater emotional ease. Curiosity promotes self-initiated exploration and mastery. In turn, this enhances learners’ confidence and sense of control, which are two critical factors known to mitigate anxiety. Student Hu’s experience offers a representative example. Previously anxious about participating in class, he began exploring English through music out of personal interest. This self-directed learning deepened his engagement with the language and significantly reduced his stress during classroom discussions. His experience illustrates how curiosity can reshape the emotional climate of language learning by reducing avoidance behaviors and building communicative confidence.

“Lately, I’ve been trying to learn more about English by myself, like reading books and watching English shows. It’s like, the more I learn because I want to, not just for school, the less I get all tense in class. For example, last month, I was studying about different types of music in English, and it was so interesting that I started listening to English songs and figuring out the lyrics. When we had a class discussion about music, I wasn’t as worried to speak up because I felt more confident. I knew I had more to share than just the stuff we learned in class.”

(Student Hu)

#### 4.3.4. The Mediating Role of Anxiety Between Curiosity and Academic Achievement

Language learning curiosity was found to indirectly enhance academic achievement by reducing foreign language anxiety. The structural model confirmed that curiosity, while influencing achievement directly, also operated through emotional regulation pathways. Specifically, students who were more curious tended to experience lower anxiety, which in turn fostered better academic outcomes. This pattern was clearly illustrated in Student Yang’s reflection. Initially overwhelmed by English-related stress, he developed a strong desire to understand unfamiliar words and linguistic structures. His intrinsic motivation redirected attention away from fear and toward active engagement. As he immersed himself in learning, his anxiety diminished, allowing for more relaxed and confident participation in class and on assessments. This case demonstrates how curiosity can act as a protective factor, transforming anxiety-inducing situations into opportunities for growth and learning.

“English used to really stress me out, especially when I didn’t understand something. But then I started getting curious about the language. When I don’t know a word, I feel that itch to look it up right then and there. When I’m so eager to learn something new, I don’t have time to be anxious. I’m too busy exploring. And it’s cool because this curiosity is like a shield against that anxiety. My grades have actually gone up because I’m not as nervous about not knowing everything. It’s like I’m more relaxed and open to learning, which I think is helping me understand better.”

(Student Yang)

## 5. Discussion

### 5.1. Junior High School Students’ Profiles of Curiosity and Anxiety in English Language Learning

This study found that junior high school students exhibited relatively high levels of language learning curiosity and moderate levels of foreign language anxiety in the context of English language learning. This suggests that students’ interest in English extended beyond academic obligations, motivating them to seek real-world exposure to the language. This aligns with Post and van der Molen (2018), who found that learners with higher levels of curiosity were more likely to pursue out-of-class learning experiences and engage actively with learning in informal settings. In contrast, foreign language anxiety was reported at a moderate level, indicating that while most students were not severely distressed, anxiety remained a persistent barrier to performance. This is consistent with findings by Bielak (2025), who noted that even moderate anxiety can impede learners’ fluency and confidence. By integrating both quantitative and qualitative data, the present study contributes a nuanced understanding of how cognitive and emotional factors coexist in adolescent language learning. It extends prior work by offering insight into the dual role of curiosity and anxiety among younger learners, highlighting the importance of fostering curiosity while simultaneously addressing anxiety in early English education.

### 5.2. Relationships Among Curiosity, Anxiety, and Academic Achievement

The results of this study revealed significant relationships among language learning curiosity, foreign language anxiety, and academic achievement. First, curiosity was positively associated with academic achievement, suggesting that students who are more inquisitive tend to engage more deeply with content, leading to better performance. This finding echoes previous research, such as Nikitina et al. (2025), which linked curiosity to sustained learning motivation in English language learning contexts. Second, anxiety was negatively related to academic achievement, indicating that heightened emotional distress can undermine cognitive focus and hinder performance. This supports findings by Sharma and Sarkar (2020), who reported that even moderate anxiety reduces learners’ efficiency in classroom settings. Third, curiosity and anxiety were negatively correlated, implying that students with higher curiosity may be less likely to experience debilitating anxiety. This pattern aligns with Berhó and Defferding (2005), who noted that curiosity can serve as a buffer against affective barriers to communication. Together, these results contribute to a more integrated understanding of how motivation and emotion interact in shaping academic outcomes, particularly among younger EFL learners, an area previously underexplored in empirical research.

### 5.3. The Mediating Role of Foreign Language Anxiety

This study found that foreign language anxiety partially mediated the relationship between language learning curiosity and academic achievement. Specifically, students who demonstrated higher levels of curiosity tended to experience reduced anxiety, which in turn facilitated better academic performance. This suggests that curiosity not only directly promotes learning but also contributes to emotional regulation, creating a more supportive internal environment for academic success. The negative path from curiosity to anxiety aligns with Vracheva et al. (2020), who reported that curiosity lowered learners’ emotional resistance and enhanced positive engagement. Likewise, the path from anxiety to academic achievement echoes findings by Vracheva et al. (2020), who observed that anxiety disrupts students’ cognitive processing and leads to lower academic performance in language contexts. By highlighting the mediating role of anxiety, this study adds a new layer to existing models of second language learning motivation, emphasizing how cognitive–emotional interactions influence academic outcomes in junior high school populations.

## 6. Conclusions

This study aimed to examine how language learning curiosity and foreign language anxiety contribute to academic achievement among junior high school students within an exam-driven educational environment. Drawing on the control-value theory of academic emotions, the findings offer empirical support for the emotional mechanisms linking motivation and learning outcomes. First, the results revealed that students generally exhibited relatively high levels of curiosity and moderate levels of anxiety, as supported by both quantitative and qualitative data. Curiosity emerged as a positive motivational factor that supports academic performance, while anxiety functioned as a negative emotional factor that hinders academic success. The identification of anxiety as a partial mediator highlights how curiosity not only enhances academic achievement directly but also indirectly by reducing emotional barriers. These insights address a gap in the literature regarding the joint role of epistemic emotions in adolescent language learning and provide a deeper understanding of how emotional and motivational factors interact during a critical stage of development.

This study contributes to a deeper understanding of how psychological factors interact to shape language learning outcomes among adolescents, offering both theoretical insights and practical guidance for language education. This study extends the control-value theory by positioning curiosity, typically regarded as a motivational trait, as a control-related cognitive resource that reduces negative emotions such as anxiety. The findings empirically support the theory’s proposition that academic emotions mediate the link between motivation and achievement, as demonstrated by the indirect effect of curiosity on performance through anxiety. By focusing on junior high school students in English as a Foreign Language classrooms, the study expands the developmental and cultural scope of the control-value theory, which has been predominantly tested in Western or tertiary education settings. Together, these insights reinforce the explanatory power of the control-value theory while also suggesting new pathways for integrating epistemic emotions such as curiosity into models of academic emotion and learning behavior.

Based on the findings of this study, several pedagogical implications can be drawn to enhance junior high school students’ English language learning experiences. First, teachers should foster curiosity by incorporating authentic and culturally engaging materials, such as English films, songs, or interactive games, which can motivate students to explore the language beyond textbook content. Second, creating a supportive and low-anxiety classroom environment is essential, as high anxiety was shown to hinder academic performance; strategies such as peer collaboration, formative assessments, and encouraging feedback can help alleviate pressure. Third, instructional design should balance cognitive challenge with learner autonomy by offering students meaningful choices in tasks or topics. This sense of control not only enhances engagement but also contributes to reduced anxiety and improved academic outcomes.

Despite its meaningful findings, this study has three main limitations. First, it employed a cross-sectional design, which restricts the ability to draw causal conclusions about the relationships among curiosity, anxiety, and academic achievement. Second, the sample was limited to junior high school students from a single region in southeastern China, which may reduce the generalizability of the results to broader or more diverse populations. Third, the study did not measure or control for the number of years students had spent learning English, which could potentially influence their emotional experiences and learning outcomes. In light of these limitations, future research should consider adopting longitudinal or experimental designs to capture how curiosity and anxiety develop over time and how they causally influence academic outcomes. Moreover, expanding the participant pool to include students from various geographic regions, grade levels, and school types could provide a more comprehensive understanding of the emotional and cognitive factors influencing language learning. Lastly, future research may include this variable to examine its role in shaping students’ curiosity, anxiety, and academic achievement more comprehensively. These improvements would enhance the external validity of the findings and offer deeper insight into the dynamic interplay of epistemic emotions and academic performance across different educational settings.

## Figures and Tables

**Table 1:** Basic information on participants invited for the interview.

Pseudonym	Gender	Grade	Curiosity	Anxiety
Zhong	Female	1	3.73	2.75
Chen	Male	2	4.18	3.25
Yang	Male	2	2.45	3.38
Feng	Female	2	4.27	4.00
Yan	Male	3	2.27	3.88
Hu	Male	3	2.73	3.25
Li	Female	3	3.82	2.88

Note: Pseudonyms were used for all participants.

**Table 2:** Different paths of variables in the SEM.

Pathway	Estimate	Standardized Estimate	S.E.	C.R.	*p*	R^2^
Curiosity→Anxiety	−0.170	−0.213	0.043	−3.914	***	0.046
Curiosity→Academic Achievement	0.353	0.381	0.044	7.970	***	0.236
Anxiety→Academic Achievement	−0.269	−0.231	0.057	−4.688	***	

Note: *** *p* < 0.001.

**Table 3:** Indirect, direct, and total effects.

Pathway	Standardized Estimate	Bias-Corrected 95% CI			Percentile 95% CI		
		Lower	Upper	*p*	Lower	Upper	*p*
Indirect Effect	0.049	0.019	0.110	0.002	0.017	0.105	0.003
Direct Effect	0.381	0.300	0.551	***	0.299	0.548	***
Total Effect	0.430	0.364	0.598	***	0.364	0.598	***

Note: *** *p* < 0.001.

## Data Availability

The data presented in this study are available upon request from the corresponding author.

## Appendix A. Items of the Curiosity and Anxiety Scales Used in This Study

### Appendix A.1. Language Learning Curiosity Scale (Adapted from Mahmoodzadeh & Khajavy, 2019)

1. I wonder how well I can speak English in contexts outside the classroom.
2. I wonder how well I can speak English when meeting a native English speaker.
3. I wonder how it would feel to speak English as fluently as a native speaker does.
4. I always like to know how to use the new words I learn in conversational situations outside the classroom.
5. I always like to learn not only the language rules but also the “exceptions”.
6. When I have a language question in mind, I cannot rest without knowing the answer.
7. I always like to learn the cultural differences between English and my native language.
8. I wonder how it would feel to speak and write English as well as my language teacher does.
9. When my language teacher corrects my grammatical mistake, I am just curious to know why it is not correct.
10. If I see or hear an unfamiliar English word, I immediately check my dictionary or ask my teacher.
11. It keeps me occupied if I cannot express what I know from my native language in English.

### Appendix A.2. Foreign Language Anxiety Scale (Short-Form Version Adapted from Botes et al., 2020)

1. Even if I am well prepared for FL class, I feel anxious about it.
2. I always feel that the other students speak the FL better than I do.
3. I can feel my heart pounding when I’m going to be called on in FL class.
4. I worry about making mistakes in FL class.
5. I can’t be confident when I speak in FL class.
6. I get nervous and confused when I am speaking in my FL class.
7. I start to panic when I have to speak without preparation in FL class.
8. It embarrasses me to volunteer answers in my FL class.
